# Balneotherapy in knee osteoarthritis: a review of therapeutic efficacy and inflammatory cytokine regulation

**DOI:** 10.1007/s00484-026-03358-3

**Published:** 2026-10-29

**Authors:** Xiangfeng Lai, Lin Ji, Yuzhe Zou, Jiong Wang, Yuanyuan Zhan, Liqing Yao, Xue Yang

**Affiliations:** 1 Department of Rehabilitation Medicine, The Second Affiliated Hospital of Kunming Medical University, Kunming, Yunnan China; 2 School of Rehabilitation, Kunming Medical University, Kunming, Yunnan China

**Keywords:** Balneotherapy, Knee osteoarthritis, Inflammatory cytokines, Signaling pathways, Mineral elements

## Abstract

Knee osteoarthritis (KOA) is a common degenerative joint disease with an inflammatory component. Balneotherapy has been used for KOA, but its clinical efficacy and mechanisms remain uncertain. This narrative review summarizes clinical evidence and proposes a hypothesis-generating mechanistic framework. PubMed and CNKI were searched up to June 2026. Interventions were categorized as mineral-water bathing, aquatic exercise/hydrotherapy, mud therapy/pelotherapy, or multimodal spa programmes, and clinical studies were assessed for risk of bias. Randomized trials and meta-analyses suggest that balneotherapy may improve short-term pain and function, with benefits reportedly persisting for 3–9 months in some studies; however, most trials had high risk of bias, heterogeneous protocols, small samples, and short follow-up. Mechanistic evidence is largely preclinical or indirect. H₂S has the most consistent anti-inflammatory and anti-catabolic rationale, acting via NF-κB and MMP-13 inhibition. Calcium and magnesium may modulate TRPV4/NLRP3 and NF-κB signaling, but human balneotherapy evidence is lacking, and mineral synergy remains unverified. A three-tier “heat–mineral–signal” framework is proposed, but target engagement after bathing and causality between biomarker changes and clinical improvement have not been established. Overall, balneotherapy may offer symptomatic benefits in KOA, but current evidence is limited and heterogeneous. The proposed mechanistic model is hypothesis-generating; higher-quality trials with standardized protocols, long-term follow-up, and integrated clinical and mechanistic outcomes are needed.

## Introduction

Knee osteoarthritis (KOA) is the most common degenerative joint disease, with an estimated 364.6 million cases worldwide in 2019 (Yang et al. 2023). In China, the China Health and Retirement Longitudinal Study (CHARLS) reported a prevalence of symptomatic KOA of 8.1% among adults aged 45 years and older, with higher rates in women (10.3%) than in men (5.7%) (Tang et al. 2016). Regional estimates across China range from approximately 10% to 20%, and obesity, female sex, and increasing age are consistent risk factors (Cui et al. 2020).

KOA is no longer viewed as a purely mechanical disease. Synovial inflammation, macrophage activation, and an imbalance between M1 and M2 macrophages contribute to pain and structural progression. Pro-inflammatory cytokines such as tumor necrosis factor-alpha (TNF-α), interleukin-1 beta (IL-1β), and interleukin-6 (IL-6) stimulate chondrocytes to release matrix metalloproteinases (MMPs), including MMP-1, MMP-3, and MMP-13, and ADAMTS-4/5, which degrade the cartilage matrix (Kuppa et al. 2022). These cytokines also recruit additional macrophages and may establish a self-reinforcing inflammatory loop.

Current non-surgical management includes education, exercise, weight management, and pharmacotherapy. However, oral non-steroidal anti-inflammatory drugs (NSAIDs) are contraindicated in some patients with cardiovascular disease or frailty (Bannuru et al. 2019), and long-term NSAID use is associated with increased cardiovascular risk (Bally et al. 2017). Most available drugs target a single pathway, which may be insufficient for the multiple pathological processes in KOA.

Balneotherapy has regained interest as a complementary, multi-component approach. It may reduce pain and improve function in some patients with KOA, with generally mild or transient adverse effects (Forestier et al. 2010; Ma et al. 2021; Protano et al. 2023). However, the term “balneotherapy” is often used broadly and may include mineral-water bathing, aquatic exercise, mud therapy, and multimodal spa programmes. These interventions differ in thermal load, mechanical loading, mineral composition, and co-interventions, and their effects cannot be attributed equally to mineral components.

This review differs from previous reviews and meta-analyses on balneotherapy for KOA and related rheumatic conditions (Ma et al. 2021; Cheleschi et al. 2022; Protano et al. 2023) in four respects. First, it distinguishes among balneotherapy modalities and specifies comparators and co-interventions. Second, it grades mechanistic claims as direct balneotherapy evidence, indirect experimental evidence, or untested hypotheses. Third, it integrates Chinese hot spring data into a global hydrochemical framework rather than treating them as a separate section. Fourth, it proposes testable research priorities rather than presenting speculative mechanisms as established causal pathways. This article is therefore framed as a hypothesis-generating narrative review, not as a systematic review or a definitive mechanistic account.

## Clinical evidence for balneotherapy in KOA

### Search strategy and selection criteria

PubMed and CNKI were searched from inception to June 2026. The search combined terms for disease (“knee osteoarthritis,” “KOA,” “osteoarthritis”), intervention (“balneotherapy,” “hydrotherapy,” “mineral water,” “thermal water,” “mud therapy,” “pelotherapy,” “hot spring,” “spa therapy”), and outcomes or mechanisms (“pain,” “function,” “WOMAC,” “VAS,” “inflammatory cytokines,” “TNF-α,” “IL-1β,” “IL-6,” “NF-κB,” “MAPK,” “NLRP3”). Reference lists of relevant reviews were also hand-searched.

- Studies were included if they met the following criteria:
- Population: adults with knee osteoarthritis.
- Intervention: balneotherapy or related modalities, categorized as mineral-water bathing, aquatic exercise/hydrotherapy, mud therapy/pelotherapy, or multimodal spa programmes.
- Comparator: tap water, no treatment, usual care, physical therapy, pharmacological treatment, or another balneotherapy modality.
- Outcomes: pain, function, quality of life, adverse events, inflammatory biomarkers, or mechanistic outcomes relevant to KOA.
- Study types: randomized controlled trials, systematic reviews or meta-analyses, prospective observational studies, and mechanistic studies in humans, animals, or cell models.
- Language: English or Chinese.

Studies were excluded if they did not involve KOA, did not evaluate balneotherapy or a related modality, reported no relevant outcome data, were conference abstracts without full text, or were duplicate publications.

This is a narrative review and was not registered as a systematic review. To improve transparency, we defined intervention categories, comparators, and criteria for assessing clinical and mechanistic evidence. Titles and abstracts were screened for relevance, and full texts were assessed against the eligibility criteria. Clinical studies were summarized in Table 1, including design, sample size, intervention, comparator, follow-up, effect estimates, and principal limitations. We distinguished within-group improvement from between-group benefit. Risk of bias was assessed using the Cochrane RoB 2 tool for randomized controlled trials and the ROBINS-I tool for non-randomized studies. No formal certainty-of-evidence grading system was applied. Mechanistic evidence was graded as:

- A: direct balneotherapy evidence in patients;
- B: indirect experimental evidence from animal, cell, donor, inhibitor, or knockout studies;
- C: untested hypothesis or speculative extrapolation.

### Intervention categories and comparators

Balneotherapy studies should not be pooled as if they tested the same intervention (Ma et al. 2021; Protano et al. 2023; Lei et al. 2024). We distinguish four categories:

1.  Mineral-water bathing: immersion in thermal or mineral water, with or without whirlpool.
2.  Aquatic exercise/hydrotherapy: exercise performed in water, in which mechanical and thermal effects are difficult to separate.
3.  Mud therapy/pelotherapy: application of mud or peloids, often combined with bathing.
4.  Multimodal spa programmes: combinations of bathing, mud, exercise, massage, and physiotherapy.

These categories differ in thermal load, mechanical loading, mineral composition, and co-interventions. Comparators vary across studies and include tap water, no treatment, usual care, physical therapy alone, and pharmacological treatment. Co-interventions such as exercise, physiotherapy, massage, and pharmacological treatment should be reported separately because they may contribute to observed effects. Clinical studies are therefore summarized in Table 1 by intervention category, comparator, co-intervention, and follow-up, and within-group improvement is distinguished from between-group benefit.

### Pain relief and functional improvement

Several RCTs suggest short-term symptom benefits, but between-group differences should be distinguished from within-group improvements. Forestier et al. (2010) reported that 50.8% of patients in the balneotherapy group achieved minimal clinically important improvement versus 36.4% in the control group (*P* = 0.005), with effects reportedly persisting for 6 months. Dilekçi et al. (2019) found that balneotherapy combined with physical therapy improved VAS pain, WOMAC, EQ-5D-3 L, and FACIT-F scores compared with physical therapy alone in elderly patients. Kulisch et al. (2014) compared Hévíz thermal mineral water with tap water and reported better VAS pain, active flexion, WOMAC physical function, and EQ-5D scores in the treatment group, with effects sustained through 15 weeks. Other trials in Table 1, including Fioravanti et al. (2010, 2012), Bálint et al. (2007), and Jiang et al. (2010), also reported improvements in pain and function, although most had high risk of bias due to lack of blinding, unclear allocation concealment, or missing data. Zheng et al. (2025) reported reduced inflammatory cytokines and lower recurrence with hot spring bathing combined with electroacupuncture and tuina, but this study was single-centre with short follow-up. These findings are encouraging but should be interpreted cautiously.

These findings are encouraging but should be interpreted cautiously. Many trials are small, blinding is difficult, and co-interventions differ. Although some trials reported between-group differences, many studies only report within-group changes. Meta-analyses of balneotherapy and hydrotherapy generally support short-term pain reduction (Lei et al. 2024; Ma et al. 2021; Protano et al. 2023; Raza 2019), but heterogeneity in spring type, temperature, immersion frequency, and treatment duration limits pooled estimates. Detailed study characteristics, effect estimates, and limitations are summarized in Table 1.

### Durability of effects

Beyond short-term pain relief and functional improvement, how long the benefits last is also clinically important. However, evidence on the durability of balneotherapy effects remains limited and heterogeneous. Harzy et al. (2009), in a systematic review of 9 RCTs including 493 patients with KOA, reported that thermal mineral water interventions improved pain and function at the end of treatment, with some improvements reportedly sustained for up to 24 weeks. Fioravanti et al. (2010) found that WOMAC improvements persisted for 9 months after daily mud therapy plus mineral baths, and analgesic consumption was reduced; however, this was a multimodal mud–bath intervention, so the specific contribution of mineral water alone cannot be isolated. Özkuk et al. (2017) compared continuous and intermittent balneotherapy schedules and observed comparable improvements between groups, but some effects diminished by 3 months; this trial lacked an untreated control group. Overall, reported durability in selected studies ranges from 3 to 9 months, but maintenance protocols have not been standardized, and evidence for recurrence prevention is scarce. Claims of long-term efficacy should therefore be restricted to the reported persistence in these studies rather than considered established durability. Follow-up durations for individual clinical trials are summarized in Table 1.

**Table 1:** Summary of clinical studies on balneotherapy for knee osteoarthritis

Study	Country	Design	*N*	Intervention	Comparator	Co-interventions	Follow-up	Main results	Main limitations	Risk of bias
Foresti-er et al. 2010	France	RCT	382	Multimodal spa therapy, 18 d	Home exercises + usual care	Home exercises; usual pharmacological treatment	6 month	MCII 50.8% vs. 36.4% (*P* = 0.005)	Zelen randomisation; treatment switching; missing data; co-interventions not controlled	High
Dilekçi et al. 2019	Turkey	RCT	305	Balneotherapy + physical therapy	Physical therapy alone	Physical therapy	Short-term	VAS, WOMAC, EQ-5D, FACIT-F, EPWORTH improved (*P* < 0.0001); responder rate 98.74% vs. 89.04%	No placebo; short follow-up; blinding difficult; allocation concealment unclear; unequal duration; no registration	High
Kulisch et al. 2014	Hungary	RCT	77	Hévíz thermal mineral water, 34 °C, 30 min, 5×/wk, 3 wk	Tap water, same protocol	Tap water, same protocol	15 week	Better VAS (*P* < 0.01), flexion, WOMAC, EQ-5D (*P* < 0.05)	No patient blinding; small single-centre; control offered free bathing after follow-up	High
Özkuk et al. 2017	Turkey	RCT	50	Peloid packs + tap water bath, 10 sessions/2 wk (continuous)	Same, 10 sessions/5 wk (intermittent)	Same as intervention	3 month	Significant clinical and QoL improvements in both groups	Small sample; short follow-up; no placebo; blinding difficult; allocation concealment unclear; missing data; no registration	High
Fioravanti et al. 2010	Italy	RCT	80	Daily mud therapy + mineral baths, 2 wk	Routine ambulatory care	Routine ambulatory care	9 month	All parameters improved; reduced analgesic consumption	No double-blind; no placebo; allocation concealment unclear; missing data with LOCF; no registration	High
Fioravanti et al. 2012	Italy	RCT	60	Sulphate–bicarbonate–calcium mineral water bath	Routine outpatient care	Routine outpatient care	12 week	VAS, Lequesne, WOMAC, SF-36 improved; reduced analgesic use	Small sample; short follow-up; no double-blind/placebo; unblinded; no registration	High
Bálint et al. 2007	Hungary	RCT	64	Nagybaracska thermal mineral water, 30 min, 5×/wk, 4 wk	Tap water, same protocol	Tap water, same protocol	3 month	WOMAC activity, pain, total improved; sustained at 3 mo	Small sample; high dropout (12/64); no ITT; allocation concealment unclear; single-centre; short follow-up; no registration	Some concerns
Wang et al. 2023	China	Non-RCT	60	Bad Ragaz Ring Method in hot spring water	Non-BRRM	Hot spring water	Short-term	Lower pain (*P* = 0.01), stiffness, function (*P* < 0.01)	Small sample; no randomisation; single centre; no follow-up; assessor unblinded; confounding not controlled	High
Wang et al. 2026	China	RCT	104	Hot spring water resistance exercise + conventional rehabilitation	Conventional rehabilitation alone	Routine rehabilitation	Short-term	Lower VAS; higher knee function, ROM, ADL (*P* < 0.05)	Single-centre; small sample; short 8-wk follow-up; allocation concealment unclear; no blinding; no registration	High
Zheng et al. 2025	China	RCT	180	Hot spring bathing + electroacupuncture + tuina	Electroacupuncture + tuina; oral drugs	Electroacupuncture; tuina; oral drugs	1 month	Lower IL-1β, IL-6, WOMAC; lower recurrence (*P* < 0.05)	No blinding; allocation concealment unclear; high dropout; no ITT; short follow-up; single centre; no registration	High
Jiang et al. 2010	China	RCT	463	Hot spring mud therapy, 42–43 °C, 25 min, 15 sessions	Whole-body thermal spring water immersion	Whole-body thermal spring water immersion, 10–15 min, 15 sessions	Short-term	Response rate 79.65% vs. 62.50% (*P* < 0.05)	Randomisation method unclear; allocation concealment unclear; no blinding; no follow-up; dropouts not reported; single centre	High

Note: *RCT* randomized controlled trial, *MCII* minimal clinically important improvement, *VAS* visual analogue scale, *WOMAC* Western Ontario and McMaster Universities Osteoarthritis Index, *EQ-5D* EuroQol 5-Dimension, *FACIT-F* Functional Assessment of Chronic Illness Therapy–Fatigue, *QoL* quality of life, *ROM* range of motion, *ADL* activities of daily living, *IL* interleukin, *LOCF* last observation carried forward, *ITT* intention-to-treat, *BRRM* Bad Ragaz Ring Method. Risk of bias, *High* lack of blinding, subjective outcomes, unclear allocation concealment, missing data, or no trial registration, *Some concerns* unclear allocation concealment, high dropout, or short follow-up. Most studies were rated High due to difficulty in blinding and reliance on patient-reported outcomes. Between-group differences should be distinguished from within-group improvements; several studies reported only within-group changes. Co-interventions varied across studies and may contribute to observed effects

### Inflammatory biomarkers and clinical benefits

Among the clinical studies in Table 1, only Zheng et al. (2025) reported inflammatory biomarker changes. Hot spring bathing combined with electroacupuncture and tuina reduced IL-1β and IL-6 and lowered recurrence, but the trial was single-centre, unblinded, had a high dropout rate, and followed patients for only 1 month. Other studies in Table 1 did not measure cytokines, so they cannot link biomarker changes to clinical improvement.

Additional biomarker studies are summarized in Table 2 (Cytokine shift). Ortega et al. (2017) reported that a 10-day pelotherapy cycle decreased serum IL-1β, TNF-α, IL-8, IL-6, and TGF-β and increased cortisol in patients with KOA. Lange et al. (2021) found that serial mud packs reduced IL-1β and increased IL-10. Silva et al. (2023) observed a significant decrease in IL-6 after a 14-day balneotherapy course. Notably, Gálvez et al. (2018) reported that serum IL-8 reduction correlated with pain relief and that TGF-β changes correlated with knee flexion improvement in elderly patients with KOA receiving whole-body hyperthermia. Ortega et al. (2017) also observed that clinical improvement paralleled decreases in multiple pro-inflammatory cytokines. These findings suggest that balneotherapy may modulate inflammatory pathways in parallel with clinical improvement.

However, these studies are small, often uncontrolled, and heterogeneous, including sulfur-containing pelotherapy and hyperthermal balneotherapy; evidence for pure mineral baths remains limited. Although some studies reported correlations between cytokine changes and clinical improvement (Gálvez et al. 2018; Ortega et al. 2017), none established causality. Therefore, the association between biomarker reduction and symptom relief should be considered hypothesis-generating rather than proof of mechanism.

### Assessment of evidence

Among the 11 clinical studies in Table 1, 10 were rated as high risk of bias and only one (Bálint et al. 2007) as “some concerns”; no study was at low risk. Common issues included lack of blinding, subjective outcomes, unclear allocation concealment, small sample sizes, short follow-up, missing data, and uncontrolled co-interventions. Given this high risk of bias and the heterogeneity across studies, the findings should be interpreted with caution, and higher-quality randomized trials with adequate blinding and long-term follow-up are needed.

## Proposed mechanistic framework: a hypothesis-generating model

No single mechanism fully explains the clinical effects of balneotherapy. We propose a three-tier framework: thermal trigger, mineral targeting, and signal-network integration. This framework is hypothesis-generating. Most mechanistic claims are indirect or have not been tested in human balneotherapy settings. The evidence supporting each mechanistic claim is summarized in Table 2.

### Thermal effects

Heat is a fundamental component of balneotherapy, but direct evidence that bathing-induced heating engages these pathways in joint tissues is limited.

1.  Heat shock proteins (HSPs). Thermal exposure induces HSP expression in experimental systems. HSPs can protect cells, but abnormal expression may also promote inflammation and chondrocyte injury (Zhao et al. 2025; Ngarmukos et al. 2020). Whether bathing-induced heating produces beneficial HSP modulation in human KOA joints remains unproven.
2.  Vasodilation and clearance of mediators. Heat increases local blood flow and may enhance clearance of metabolic waste products (Iantomasi et al. 2025; Stuhlmeier 2009). However, this has not been directly demonstrated in KOA joints after bathing.
3.  TRPV1/CGRP modulation. Thermal stimulation may desensitize transient receptor potential vanilloid 1 (TRPV1) channels and reduce the release of calcitonin gene-related peptide (CGRP) and substance P (Tominaga et al. 1998; Russell et al. 2014). This remains an indirect mechanism, and whether balneotherapy produces this effect in human KOA joints is unknown.

Overall, evidence for thermal mechanisms is mainly indirect or preclinical; direct evidence from human KOA joint tissues after balneotherapy is limited.

### Mineral-specific effects

Minerals and ions in thermal water may exert local or systemic effects through percutaneous absorption or interaction with skin receptors. However, the stratum corneum has low permeability to ionic minerals, and the concentrations reaching joint tissues after bathing are unknown. Therefore, most mineral-specific claims should be regarded as indirect or hypothetical. The evidence supporting each mineral-specific mechanism is summarized in Table 2.

#### Sulfur/H₂S

H₂S donors have shown anti-inflammatory and anti-catabolic effects in osteoarthritis chondrocyte models, including inhibition of NF-κB and reduction of MMP-13, IL-6, and PGE-2 (Magro et al. 2018). Endogenous H₂S production via 3-mercaptopyruvate sulfurtransferase (3-MST) is downregulated in human osteoarthritis cartilage, and 3-MST knockout mice show aggravated joint calcification and osteoarthritis severity (Nasi et al. 2020). Sodium hydrosulfide (NaHS) partially inhibits IL-1β-induced MAPK activation in synovial fibroblasts (Sieghart et al. 2015). These are indirect preclinical findings. Whether sulfur bathing delivers H₂S to joint tissues at active concentrations is unknown, and direct balneotherapy evidence in patients is lacking.

#### Calcium

Calcium-sensing receptor (CaSR) and TRPV4 have been implicated in chondrocyte differentiation and macrophage polarization. Sun et al. (2022) reported that TRPV4 inhibition reduced M1 macrophage polarization and NLRP3 expression in a rat osteoarthritis model. Zhang et al. (2019) linked fluid shear stress and CaSR to chondrocyte terminal differentiation. These findings do not demonstrate that calcium in thermal water reaches joint tissues or modulates TRPV4 in vivo. Calcium may even promote M1 polarization under some conditions, and the net effect on the synovial microenvironment is unknown. Direct human balneotherapy evidence is lacking.

#### Magnesium

Magnesium deficiency increases pro-inflammatory cytokines, whereas magnesium supplementation inhibits NF-κB nuclear translocation in macrophages (Hu et al. 2018). Wang et al. (2024) reported that 5 mmol/L Mg²⁺ suppresses NLRP3 inflammasome activation. Notably, 5 mmol/L Mg²⁺ corresponds to approximately 121.5 mg/L elemental magnesium. This differs from the 500 mg/L commonly cited in balneotherapy contexts, which typically refers to water hardness rather than magnesium concentration. Human clinical validation under real balneotherapy conditions is still lacking.

#### Composite minerals and synergy

Composite mineral waters have been studied in RCTs and animal models (Bálint et al. 2007; Fioravanti et al. 2012, 2013; Kim et al. 2020). These studies suggest possible benefits, but they cannot establish mineral synergy because thermal effects, mud, exercise, and co-interventions are not controlled. The proposed calcium/magnesium ratio effect on NLRP3 remains an untested hypothesis; no direct evidence shows that bathing-derived Ca²⁺/Mg²⁺ ratios modulate NLRP3 in human joint tissues.

#### Percutaneous absorption of minerals

A critical caveat is that the stratum corneum has low permeability to ionic minerals, and the amount absorbed percutaneously into the systemic circulation is far below the millimolar concentrations used in in vitro experiments. Cheleschi et al. (2022) noted that the mechanisms of mineral absorption through the skin remain not fully elucidated. Therefore, mineral effects are unlikely to be primarily mediated by systemic absorption. Alternative pathways may include skin–neural reflex arcs, in which bioactive substances in thermal baths stimulate mechanical, thermal, pressure, and chemical receptors, thereby exerting neuroreflexive and humoral effects (Rapolienė et al. 2016). Future studies should measure local mineral concentrations in synovial fluid rather than relying solely on serum levels, in order to distinguish systemic absorption from local or neurally mediated effects.

### Convergent signaling: NF-κB, MAPK, and NLRP3

NF-κB, MAPK, and NLRP3 are plausible convergence points, but evidence that balneotherapy engages these pathways in human joint tissues is indirect.

NF-κB is an integrative signaling hub linking mechanical stress, innate immunity, and metabolic signals in osteoarthritis (Wan and Zheng 2026). Hyperthermia may block p38 MAPK activation in experimental systems (Stuhlmeier 2009), and H₂S donors inhibit NF-κB and MAPK in preclinical models (Magro et al. 2018; Sieghart et al. 2015). Aberrant MAPK signaling, including ERK, p38, and JNK, drives inflammation and cartilage degradation (Fan et al. 2025). Magnesium inhibits NF-κB nuclear translocation and NLRP3 activation in cell and animal models (Hu et al. 2018; Wang et al. 2024). Calcium may activate NLRP3 via TRPV4/ROS signaling (Sun et al. 2022), and NLRP3 activation drives M1 macrophage polarization.

**Fig. 1:**
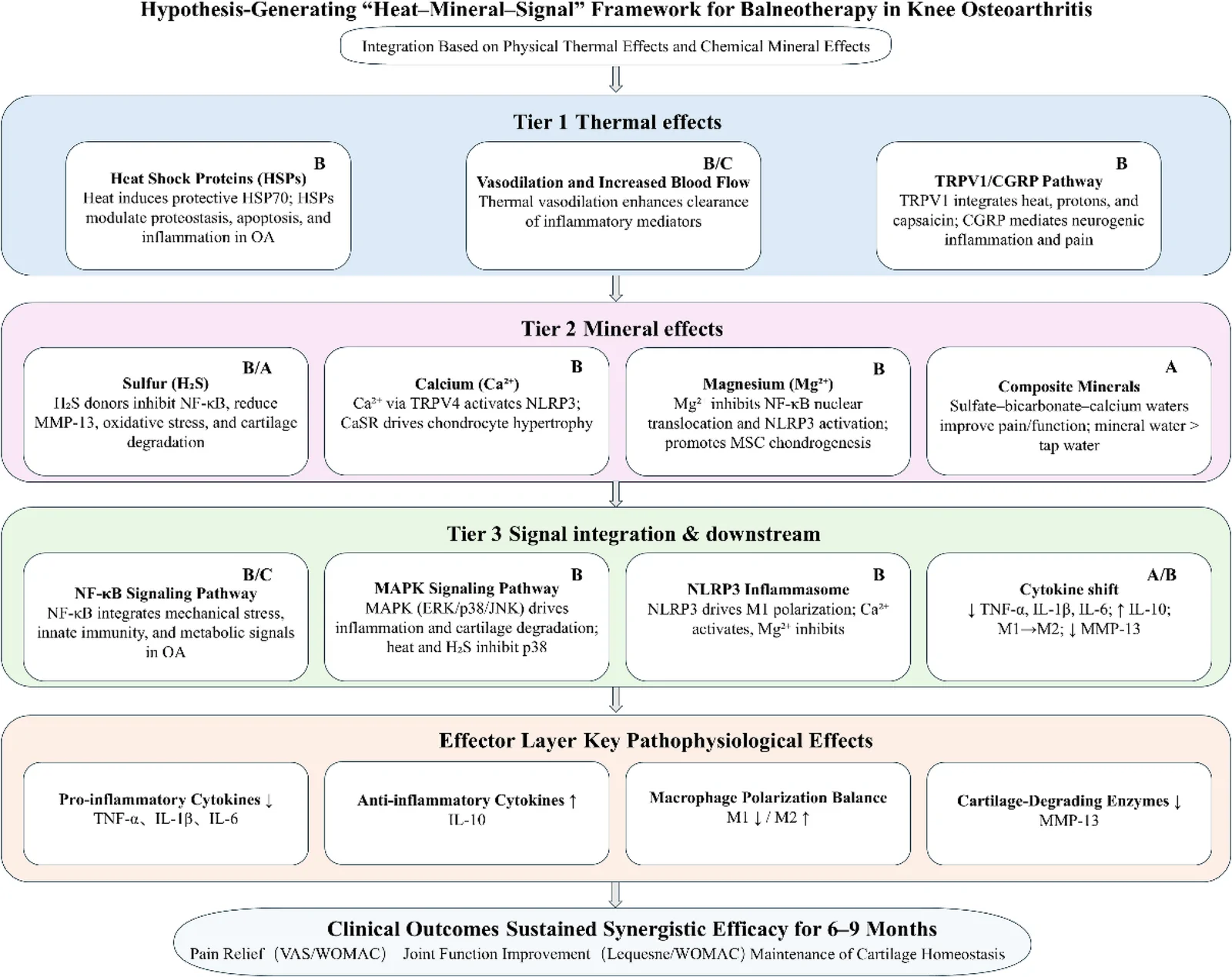
Hypothesis-generating “heat–mineral–signal” framework for balneotherapy in knee osteoarthritis. The framework integrates physical thermal effects (Tier 1), chemical mineral effects (Tier 2), and convergent molecular signaling (Tier 3) to produce downstream pathophysiological effects and clinical outcomes. The letters A, B, and C indicate the strength of evidence supporting each component, as defined in Table 2 (A = direct balneotherapy evidence in patients; B = indirect experimental evidence; C = untested hypothesis). Solid arrows indicate mechanisms supported by existing experimental or clinical literature, whereas the overall complete causal chain from bathing to target engagement and cartilage protection remains partly hypothetical and requires further validation in human trials. Clinical benefits shown are symptom-based (pain relief and functional improvement); cartilage protection remains unproven

However, whether bathing delivers these minerals to joint tissues at active concentrations is unknown, and the relative contribution of thermal, mineral, and mechanical effects cannot be separated in most clinical studies. Downstream cytokine shifts reported in clinical studies are summarized in Table 2. The proposed “heat–mineral–signal” model is therefore largely an extrapolation. The complete causal chain from bathing to target engagement to clinical benefit has not been demonstrated. This framework should be regarded as hypothesis-generating rather than established (Fig. 1; Table 2).

**Table 2:** Mechanistic evidence supporting the “heat–mineral–signal” model in knee osteoarthritis

Tier / Component	Mechanism claim	Key evidence	Evidence type	Status
Tier 1: Thermal effects				
Heat → HSPs	Heat induces protective HSP70; HSPs modulate proteostasis, apoptosis, and inflammation	Zhao et al. 2025; Ngarmukos et al. 2020	B	Plausible
Heat → TRPV1/CGRP	TRPV1 integrates heat, protons, and capsaicin; CGRP mediates neurogenic inflammation and pain	Tominaga et al. 1998; Russell et al. 2014	B	Plausible
Heat → vasodilation & clearance	Thermal vasodilation enhances clearance of inflammatory mediators	Iantomasi et al. 2025; Stuhlmeier 2009	B/C	Plausible
Tier 2: Mineral effects				
H₂S → NF-κB ↓, MMP-13 ↓	H₂S donors inhibit NF-κB, reduce MMP-13, oxidative stress, and cartilage degradation	Magro et al. 2018; Sieghart et al. 2015; Nasi et al. 2020; Benedetti et al. 2010	B/A	Plausible
Ca²⁺ → TRPV4/NLRP3 (bidirectional)	Ca²⁺ via TRPV4 activates NLRP3; CaSR drives chondrocyte hypertrophy	Sun et al. 2022; Zhang et al. 2019	B	Plausible
Mg²⁺ → NF-κB ↓, NLRP3 ↓	Mg²⁺ inhibits NF-κB nuclear translocation and NLRP3 activation; promotes MSC chondrogenesis	Hu et al. 2018; Wang et al. 2024	B	Plausible
Composite minerals → synergy	Sulfate–bicarbonate–calcium waters improve pain/function; mineral water > tap water	Fioravanti et al. 2012; Bálint et al. 2007; Kulisch et al. 2014	A	Plausible
Tier 3: Signal integration & downstream				
NF-κB hub	NF-κB integrates mechanical stress, innate immunity, and metabolic signals in OA	Wan and Zheng 2026	B/C	Plausible
MAPK pathway	MAPK (ERK/p38/JNK) drives inflammation and cartilage degradation; heat and H₂S inhibit p38	Fan et al. 2025; Stuhlmeier 2009; Sieghart et al. 2015	B	Plausible
NLRP3 integration	NLRP3 drives M1 polarization; Ca²⁺ activates, Mg²⁺ inhibits	Sun et al. 2022; Wang et al. 2024	B	Plausible
Cytokine shift	↓ TNF-α, IL-1β, IL-6; ↑ IL-10; M1→M2; ↓ MMP-13	Ortega et al. 2017; Lange et al. 2021; Silva et al. 2023; Gálvez et al. 2018	A/B	Plausible

Note: Evidence type: A direct balneotherapy evidence in patients (RCT or systematic review/meta-analysis), *B* indirect experimental evidence from animal, cell, donor, inhibitor, or knockout studies, *C* untested hypothesis or speculative extrapolation. B/A or B/C indicates mixed evidence. Status: “Plausible” = supported by indirect or partial evidence but not yet confirmed by large-scale human trials. Key evidence lists representative sources, not an exhaustive list. Abbreviations: *HSPs* heat shock proteins, *TRPV1* transient receptor potential vanilloid 1, *CGRP* calcitonin gene-related peptide, *NF-κB* nuclear factor kappa B, *MAPK* mitogen-activated protein kinase, *NLRP3* NOD-like receptor family pyrin domain containing 3, *H₂S* hydrogen sulfide, *MMP-13* matrix metalloproteinase-13, *CaSR* calcium-sensing receptor, *TRPV4* transient receptor potential vanilloid 4, *MSC* mesenchymal stem cell, *ROS* reactive oxygen species, *ERK* extracellular signal-regulated kinase, *p38* p38 mitogen-activated protein kinase, *JNK* c-Jun N-terminal kinase

## Global hydrochemical diversity and implications for mechanism-informed research

Hot springs differ globally in mineral composition. European springs have been classified as saline, alkaline, sulfurous, calcareous, ferruginous, and low-mineralization (Corwin 1930), whereas Chinese springs commonly include sulfur, bicarbonate, and chloride types (Najar et al. 2022; Hao et al. 2024; Qin et al. 2025). These hydrochemical differences may inform mechanism-oriented research, but they do not by themselves establish therapeutic specificity or mineral synergy. The mineral-specific mechanistic evidence is summarized in Table 2.

Studies from China illustrate both the opportunities and limitations of this approach. Among the clinical studies summarized in Table 1, Wang et al. (2023) reported that the Bad Ragaz Ring Method in hot spring water was associated with a greater reduction in WOMAC total score from baseline than non-BRRM treatment, although this was a prospective observational study. Wang et al. (2026) reported that hot spring water resistance exercise improved VAS, Lysholm scores, range of motion, and ADL scores compared with conventional rehabilitation. Zheng et al. (2025) reported that hot spring bathing combined with electroacupuncture and tuina reduced VAS scores and inflammatory cytokines and lowered recurrence compared with oral drugs or electroacupuncture/tuina alone. Jiang et al. (2010) reported a higher response rate with hot spring mud therapy than with whole-body immersion, but the randomization method was not clearly reported.

These studies are promising but mostly small, regional, and published in Chinese-language journals. They should be interpreted within the global discussion of sulfur and bicarbonate springs rather than as a separate national literature. Head-to-head trials comparing springs with different mineral compositions, with standardized protocols and co-interventions, are needed to determine whether specific minerals contribute to clinical effects.

## Challenges and research agenda

### Clinical and methodological challenges

Current trials often have small samples, difficult blinding, and heterogeneous protocols. As summarized in Table 1, most clinical studies were rated as high risk of bias due to these issues. Standardized protocols covering temperature, duration, frequency, total sessions, and co-interventions are lacking, and the position of balneotherapy in the KOA treatment algorithm remains unclear. Future trials should use assessor-blinded designs, objective endpoints, and explicit MCII thresholds. They should also clearly define intervention categories, distinguishing mineral-water bathing from aquatic exercise, mud therapy, and multimodal spa programmes.

### Material and mechanistic challenges

Head-to-head RCTs comparing springs with distinct mineral compositions remain limited. Most studies report “thermal spring water” generically without systematic hydrochemical analysis, and quantitative data on percutaneous absorption and synovial fluid mineral concentrations are scarce. The complete causal chain from bathing to joint inflammation and cartilage protection has not been elucidated, as summarized in Table 2. Anti-inflammatory biomarker evidence is predominantly derived from sulfur-containing pelotherapy, and direct evidence for pure mineral baths remains limited. Future research should combine hydrochemical analysis with clinical outcomes, use component-specific interventions, and apply multi-omics and quantitative exposure assessment.

### Long-term follow-up

Evidence on long-term maintenance and recurrence prevention remains limited. As summarized in Table 1, the longest follow-up among the included clinical studies was 9 months, and no study evaluated maintenance protocols or recurrence rates beyond that period. Future studies should include long-term follow-up (≥ 12 months) and define optimal maintenance regimens, including frequency, interval, and duration.

### Limitations of this review

This is a narrative review and was not registered. Selection and publication bias cannot be excluded. The literature search was limited to PubMed and CNKI, so relevant studies in other databases or grey literature may have been missed. Included studies were heterogeneous in design, mineral composition, treatment duration, and outcome measures. Some trials had small samples, lacked blinding, or had short follow-up. Much of the mechanistic evidence comes from in vitro or animal studies, and the association between cytokine changes and clinical improvement should not be interpreted as proven causality. In addition, the Raza (2019) meta-analysis was a master’s thesis rather than a peer-reviewed systematic review; its findings should therefore be interpreted with caution.

## Conclusion

As summarized in Table 1, some randomized trials and systematic reviews suggest that balneotherapy may improve pain and function in KOA, with benefits reportedly persisting for 6 to 9 months in some studies. However, the evidence is heterogeneous, and different modalities (mineral-water bathing, aquatic exercise, mud therapy, and multimodal spa programmes) should not be equated. As summarized in Table 2, mechanistic evidence is mainly indirect or preclinical. H₂S has the most consistent preclinical rationale, whereas calcium and magnesium effects on TRPV4/NLRP3, Ca²⁺/Mg²⁺ ratio effects, mineral synergy, and cartilage protection remain unverified. The proposed heat–mineral–signal framework is hypothesis-generating and has not been experimentally validated in patients. No disease-modifying or cartilage-protective effect has been demonstrated. Future work should separate modalities, standardize protocols, assess evidence systematically, and link mineral composition to molecular targets and clinical outcomes.

## Data Availability

No datasets were generated or analysed during the current study.

## Abbreviations

KOA: Knee Osteoarthritis
TNFα: Tumor Necrosis Factor alpha
IL 1β: Interleukin 1beta
IL‑6: Interleukin‑6
IL‑10: Interleukin‑10
NF‑κB: Nuclear Factor kappa B
MAPK: Mitogen‑Activated Protein Kinase
H₂S: Hydrogen Sulfide
TRPV4: Transient Receptor Potential Vanilloid 4
NLRP3: NOD‑like Receptor Family Pyrin Domain Containing 3
MMP‑13: Matrix Metalloproteinase‑13
CHARLS: China Health and Retirement Longitudinal Study
BMI: Body Mass Index
MMP‑1: Matrix Metalloproteinase‑1
MMP‑3: Matrix Metalloproteinase‑3
ADAMTS‑4/5: A Disintegrin and Metalloproteinase with Thrombospondin Motifs 4/5
IL‑8: Interleukin‑8
CCL2/3/5: C‑C Motif Chemokine Ligand 2/3/5
MMP‑9: Matrix Metalloproteinase‑9
COMP: Cartilage Oligomeric Matrix Protein
M1/M2: Macrophage Polarization Phenotypes M1/M2
NSAIDs: Non‑Steroidal Anti‑Inflammatory Drugs
OARSI: Osteoarthritis Research Society International
RCT: Randomized Controlled Trial
MCII: Minimal Clinically Important Improvement
VAS: Visual Analogue Scale
WOMAC: Western Ontario and McMaster Universities Osteoarthritis Index
EQ‑5D‑3L: EuroQol 5‑Dimension 3‑Level
FACIT‑F: Functional Assessment of Chronic Illness Therapy‑Fatigue
WMD: Weighted Mean Difference
SMD: Standardized Mean Difference
TGF‑β: Transforming Growth Factor‑beta
HPA: Hypothalamic‑Pituitary‑Adrenal Axis
OA: Osteoarthritis
HSP: Heat Shock Protein
HSP70: Heat Shock Protein 70
HSPA4L: Heat Shock Protein Family A Member 4‑Like
METTL3: Methyltransferase‑like 3
m6A: N6‑methyladenosine
TRPV1: Transient Receptor Potential Vanilloid 1
CGRP: Calcitonin Gene‑Related Peptide
3‑MST: 3‑Mercaptopyruvate Sulfurtransferase
CaSR: Calcium‑Sensing Receptor
NO: Nitric Oxide
PGE‑2: Prostaglandin E2
ROS: Reactive Oxygen Species
JNK: c‑Jun N‑terminal Kinase
ERK: Extracellular Signal‑Regulated Kinase
Akt1/2: Protein Kinase B 1/2
BRRM: Bad Ragaz Ring Method
ADL: Activities of Daily Living
